# The epigenetic mechanisms involved in the treatment resistance of glioblastoma

**DOI:** 10.20517/cdr.2024.157

**Published:** 2025-03-13

**Authors:** Aanya Shahani, Hasan Slika, Ahmad Elbeltagy, Alexandra Lee, Christopher Peters, Toriyn Dotson, Divyaansh Raj, Betty Tyler

**Affiliations:** Hunterian Neurosurgical Laboratory, Department of Neurosurgery, Johns Hopkins University School of Medicine, Baltimore, MD 21231, USA.

**Keywords:** DNA methylation, epigenetics, glioblastoma, histone modification, miRNA, treatment resistance, tumoral heterogeneity

## Abstract

Glioblastoma (GBM) is an aggressive malignant brain tumor with almost inevitable recurrence despite multimodal management with surgical resection and radio-chemotherapy. While several genetic, proteomic, cellular, and anatomic factors interplay to drive recurrence and promote treatment resistance, the epigenetic component remains among the most versatile and heterogeneous of these factors. Herein, the epigenetic landscape of GBM refers to a myriad of modifications and processes that can alter gene expression without altering the genetic code of cancer cells. These processes encompass DNA methylation, histone modification, chromatin remodeling, and non-coding RNA molecules, all of which have been found to be implicated in augmenting the tumor’s aggressive behavior and driving its resistance to therapeutics. This review aims to delve into the underlying interactions that mediate this role for each of these epigenetic components. Further, it discusses the two-way relationship between epigenetic modifications and tumor heterogeneity and plasticity, which are crucial to effectively treat GBM. Finally, we build on the previous characterization of epigenetic modifications and interactions to explore specific targets that have been investigated for the development of promising therapeutic agents.

## INTRODUCTION

The most common primary malignant brain tumor is glioblastoma (GBM). Approximately, around 13,000 individuals are diagnosed with GBM every year in the United States^[1]^. GBM is characterized by its aggressive nature and ability to recur. In this context, the 5-year survival rate of patients remains below 7% despite multidisciplinary management with surgical resection, radiation therapy, and chemotherapy^[1]^. Hence, the tumor’s resistance to treatment remains a major concern and a topic of substantial research interest. This resistance is a multifaceted process with several underlying mechanisms. One of the major contributors to the development and maintenance of treatment resistance is the epigenetic profile of GBM cells, which is defined as the set of alterations and effectors that regulate the expression of genes and the resultant phenotype of cells without changing their DNA sequence^[2,3]^. These epigenetic processes include DNA methylation and demethylation, histone modifications, chromatin remodeling, and non-coding RNAs. Each of these modifications, along with the enzymes and molecular pathways that interact with them, have been shown to be implicated in driving treatment resistance in at least one mechanism. This review aims to highlight the significant implication of epigenetic alterations and processes in GBM’s resistance to therapeutic modalities. It also discusses the heterogeneity and plasticity present within GBM tumors, which include the diverse epigenetic profiles. Finally, the review builds on these aspects to explore targeted agents and therapeutic modalities that have been shown to interfere with the epigenetic profile of GBM tumors and recircuit it in a way that attenuates treatment resistance, augments the efficacy of existing therapeutics, and potentially could improve patient outcomes.

## EPIGENETIC PROCESSES IMPACT GLIOBLASTOMA RESISTANCE TO TREATMENT

### DNA methylation

DNA methylation, initially documented in 1948, stands as one of the most extensively researched epigenetic modifications. In humans, this process involves the attachment of a methyl group to cytosine residues, primarily at CpG sites, facilitated by a group of enzymes known as DNA methyltransferases (DNMTs). One-carbon metabolism provides the methyl groups necessary for the methylation of cytosine residues in DNA, particularly in CpG islands, influencing gene expression and chromatin structure. Analysis of glioblastoma surgical specimens from both initial presentation and recurrence shows that enzymes involved in one-carbon (1-C) purine synthesis are upregulated in recurrent glioblastoma. Furthermore, higher expression of these enzymes is associated with a shorter time to tumor recurrence^[4]^. DNA methylation serves several pivotal roles across various stages of human development and throughout life, including transcriptional regulation, genomic imprinting, preservation of X-chromosome inactivation, chromosomal maintenance, and upkeeping genomic stability^[5-7]^. Aberrant DNA methylation, observed in cancer cells, involves genome-wide hypomethylation and site-specific hypermethylation, primarily affecting CpG islands located within gene expression regulatory regions. These alterations play a significant role in tumor initiation, advancement, and resistance to treatment^[8]^. In addition to the more conventionally explored CpG island methylation, another form of non-CpG DNA methylation has been investigated. This form of methylation is known as CpH (where H refers to any base other than G). Herein, CpH methylation especially occurs in the brain^[9]^ and has been associated with alpha-synuclein expression in Parkinson’s disease^[10]^ and with risk loci in Schizophrenia^[11]^. However, to date, the impact of CpH methylation has not been explored in the context of brain tumors and its potential association with oncogenic gene expression and aggressive phenotypes in these tumors.

**Figure 1:**
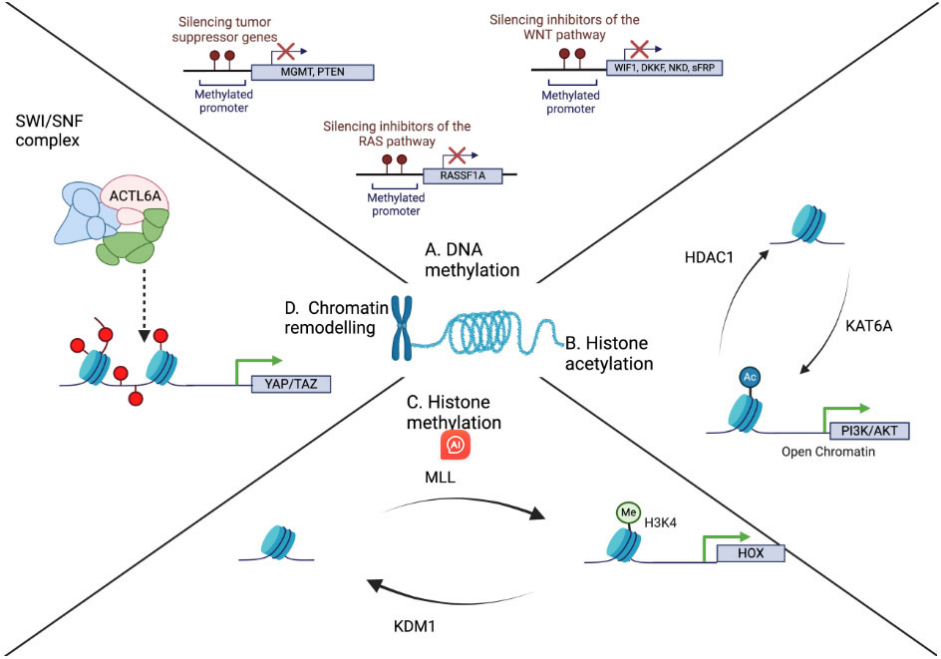
Illustration of the different epigenetic modifications that target chromatin and contribute to treatment resistance in GBM. (A) DNA methylation silences the promoters of tumor suppressor genes, hence contributing to the proliferative ability of GBM cells. This mechanism also has the ability to suppress the expression of inhibitors of the WNT pathway (WIF1, DKKF, NKD, sFRP) and RAS pathway (*RASSF1A*); (B) Histone acetylation, which is achieved by histone acetylases and reversed by histone deacetylases, can also contribute to the regulation of chromatin structure and gene expression. For example, histone acetylation by KAT6A can lead to increased expression of the genes involved in the overactivation of the PI3K/AKT oncogenic pathway. This is reversed by the histone deacetylase HDAC1; (C) Histone methylation is another alteration that can control the expression of genes. For instance, the interplay between the histone methylase MLL and the demethylase KDM1 can regulate the expression of HOX genes, which are implicated in cancer proliferation and treatment resistance; (D) The chromatin remodeling complex SWI/SNF can alter the architecture of chromatin through several of its domains. One such domain, ACTL6A, can promote the expression of the YAP/TAZ pathway, which, in turn, contributes to treatment resistance. GBM: Glioblastoma multiforme; *MGMT*: methylguanine methyltransferase; *PTEN: phosphatase and tensin homolog*; WNT: wingless; WIF: WNT inhibitory factor; DKKF: dickkopf; NKD: naked cuticle; sFRP: secreted frizzled-related protein family; RASSF1: ras association domain family; HDAC: histone deacetylase; KAT: lysine acyltransferase; MLL: mixed lineage leukemia; KDM: histone lysine demethylase; SWI/SNF: switch/sucrose non-fermentable; ACTL6A: actin-like protein 6A; PI3K/AKT: phosphoinositide-3-kinase-protein kinase B; HOX: homeobox; YAP/TAZ: yes-associated protein/transcriptional co-activator with PDZ-binding motif.

In the context of GBM, DNA methylation frequently targets the promoter regions of tumor suppressor genes, which are responsible for restraining cell growth, division, and promoting programmed cell death. When these genes undergo silencing via DNA methylation, their ability to suppress tumor growth is compromised, leading to uncontrolled proliferation of tumor cells. Examples of tumor suppressor genes affected by DNA methylation in GBM include *Phosphatase and tensin homolog* (*PTEN*) and *O6-methylguanine-DNA methyltransferase* (*MGMT*)^[12,13]^ [Figure 1A]. In GBM, this DNA methylation disrupts transcription factors binding to the promoter region, thus inhibiting the initiation of gene expression^[14]^.

The *MGMT* gene encodes the DNA-repair protein O6-alkylguanine-DNA-alkyltransferase (AGT)^[15]^. AGT plays a crucial role in cellular physiology by removing alkylating lesions at the O6 position of guanine, thereby maintaining genomic stability and preventing DNA damage, including that induced by alkylating chemotherapeutic agents such as temozolomide (TMZ)^[16]^. *MGMT* expression varies widely among different types of tumors and normal tissues. Higher levels of *MGMT* expression contribute to increased DNA stability and protect cells from the deleterious effects of alkylating agents used in chemotherapy^[15]^. Conversely, decreased or absent *MGMT* expression increases the susceptibility to chemotherapeutic agents and enhances sensitivity to alkylating agents^[15]^. In tumors, *MGMT* promoter methylation effectively silences *MGMT* expression, leading to increased responsiveness to chemotherapy that includes alkylating agents^[17]^. In the clinical setting, in GBM, *MGMT* promoter methylation status helps predict the efficacy of TMZ therapy, since patients with *MGMT* promoter methylated tumors tend to have a better response to TMZ chemotherapy and longer overall survival compared to those without promoter methylation^[18,19]^.

Moreover, DNA methylation can impact genes involved in the phosphatidylinositol-3-kinase (PI3K)/protein kinase B (Akt)/mammalian target of rapamycin (mTOR) pathway, which is frequently dysregulated in GBM, altering the sensitivity of tumor cells to targeted therapies [Figure 1B]. In general, the phosphoinositide-3-kinase-protein kinase B (PI3K-AKT) signaling pathway promotes cellular proliferation and growth while concurrently suppressing apoptosis^[20]^. Singh *et al.* demonstrated that depletion of TP53, *PTEN*, and NF1 in human brain organoids induces a glioma-like phenotype *in vitro*, underscoring the significance of *PTEN* suppression in gliomas^[21]^. Wiencke *et al.* showed that methylation of the *PTEN* promoter is associated with phosphorylation of protein kinase B (PKB/Akt), indicating functional activation of the PI3K pathway^[22]^. This activation contributes to the promotion of GBM growth and enhancement of cell survival.

Hes Related Family BHLH Transcription Factor with YRPW Motif 1 (HEY1) belongs to the Hairy/Enhancer of split [H/E(spl)] family of basic helix-loop-helix transcription factors^[23]^. It plays a crucial role in sustaining neural precursor cells following Notch signaling. The Notch receptor undergoes cleavage by TNF-α-converting enzyme and γ-secretase, resulting in the generation of the active Notch intracellular domain (NICD)^[24]^. NICD translocates to the nucleus, where it facilitates the expression of Notch targets such as HEY1 and hairy and enhancer of split-1 (HES1)^[25]^. Notch signaling is essential in GBM. Notch receptors and their ligands are markedly upregulated in GBM, indicating abnormal activation of Notch signaling^[26]^. Elevated levels of Notch1 and NICD1 are frequently observed in GBM. Increased activation of Notch1-mediated signaling contributes to the development and resistance to chemotherapy in GBM^[24,27-29]^. Hence, targeting the Notch pathway has emerged as a promising approach for future GBM therapies^[24]^. HEY1 expression rises proportionally with the severity of astrocytoma tumor grades and is associated with both reduced overall survival and reduced disease-free survival rates^[23]^. Studies, such as that by Tsung *et al.*, have shown that the methylation status of HEY1 plays a role in GBM pathogenesis and can serve as a predictive marker for GBM patients^[30]^.

In GBM, aberrant promoter methylation contributes to the activation of the Wingless (WNT) pathway by silencing various negative regulators. These include genes encoding WNT inhibitory factor 1 (WIF1)*,* members of the secreted frizzled-related protein (sFRP) family, Dickkopf (DKKF), and naked cuticle (NKD) family members^[31,32]^. Likewise, promoter methylation also silences negative regulators of the Ras pathway, such as the Ras association *(RalGDS/AF-6)* domain family member *RASSF1A*^[33]^ [Figure 1].

### Histone modification

The role of histone post-translational modifications (PTMs) in regulating gene expression is well recognized^[34]^. This is primarily accomplished by altering the structure and/or function of chromatin, an octamer core consisting of paired copies of histone proteins H2A, H2B, H3, and H4 enveloped by double-stranded DNA^[35]^. Attached to these histones are N-terminal tails, which extend beyond the nucleosome core and are thus susceptible to PTMs, including methylation, acetylation, phosphorylation, and ubiquitination. Abnormalities within these histone modifications have been demonstrated to contribute to gene transcription, resulting in increased GBM proliferation, invasion, and ultimately therapeutic resistance^[36-39]^.

#### Histone methylation and demethylation

Methylation of histone tails occurs because of the actions of histone methyltransferases (HMTs), which catalyze the transfer of methyl groups from S-adenosylmethionine onto the fundamental residues of a histone tail^[40]^. Methylation most commonly occurs on the side-chain nitrogen atoms of arginines and lysines^[41]^ [Figure 1C]. Accordingly, HMTs are generally divided into lysine methylating proteins (KMTs), such as SET [Su(var), Enhancer of Zeste, and Trithorax] domain-containing and DOT1-like proteins, and arginine methylating proteins such as the arginine N-methyltransferases (PRMTs)^[41]^. Unlike acetylation, methylation alters the hydrophobicity and hydrogen binding radii of methyl-lysine, thereby modifying the binding properties of these sites without neutralizing the charge of the target residue^[42]^. Reversal of histone methylation is catalyzed by histone demethylases. Similar to their methylating counterparts, demethylases are segregated between lysine demethylation enzymes (KDMs), such as amine oxidase domain-containing (LSD) proteins and Jumonji C (JmjC) domain-containing proteins, while identifying arginine-selective demethylases has proven generally elusive^[43,44]^.

In humans, histone H3 is arguably the most cited due to its highly conserved sequence in eukaryotic organisms, which enables the identification of numerous PTMs on the histone^[45]^. Particularly, methylation occurs on the lysine residue sites of H3, with H3K4, H3K36, and H3K79 methylations marking transcriptionally active genes and H3K9, H4K20, and H3K27 methylations signaling inactive genes^[46-48]^. For example, it is understood that H3K4 is methylated by the mixed lineage leukemia (MLL) family of proteins in GBM biology-leading to upregulation of genes implicated in differentiation and self-renewal [Figure 1C]^[46,49]^. Furthermore, MLL has been shown to directly increase the GBM stem-like cell (GSC) proliferation rate *in vitro* and *in vivo* through activation of the HOXA10 transcription factor and subsequent upregulation of developmental Hox genes^[50]^. Another example is enhancer of Zeste homolog 2 (EZH2), the primary HMT enzyme for H3K27me methylation, which has been increasingly aligned with GBM tumorigenicity, stemness, and resistance to therapeutics such as TMZ through its upregulation of c-MYC expression and STAT3 phosphorylation^[51-57]^. Moreover, Su(var)3-9/enhancer-of-zeste/trithorax (SET) domain and mariner transposase fusion gene (SEMTAR), an additional HMT responsible for H3K36 methylation, has been highlighted as a contributor to GBM radiation resistance through its recruitment of Ku80, a DNA damage repair protein^[57]^. Although mechanistic pathways for HMT influence are generally unclear, it is evident that they present unique targets for potential epigenetic treatment avenues. Similarly important to GBM tumorigenicity is the H3K9 family of methyltransferases. Euchromatic histone lysine methyltransferase 2 (EHMT2) expression has been linked to a pro-tumorigenic effect^[58,59]^, with *in vitro* inhibition reducing overall H3K9 dimethylation and increasing c-MYC-dependent autophagy and autophagy-dependent differentiation. Additionally, EHMT2 methylation of hypoxia-inducible factor-1 (HIF-1) inhibited hypoxia adaptation and cellular invasion in the U251MG cell line, ultimately revealing a hypoxia-induced mechanism of negative feedback for HIF-1 activity and human GBM cell mobility^[60,61]^. Similarly, methylation of H3K9 by Suv39H1 and SETDB1 has demonstrated decreased expression of the gene. Further studies in GBM have noted an increased expression of both Suv39H1 and SETDB1 as compared to normal brains, with short-hairpin-RNA-mediated (shRNA) knockdown of SETDB1or chaetocin-mediated inhibition of Suv39H1 being associated with increased apoptosis and reduced migration and colony formation in T98G and GOS-3 glioma cell lines^[62-65]^.

Generally, however, histone lysine methylation serves to modify the regulation of transcription and chromatin structure dependent on the degree and site of methylation. Notably, this includes the association of H3K4 monomethylation with enhancer regions, di- and trimethylation of H3K4 with promoter regions and transcription start sites, and trimethylation of H3K36 in gene bodies of actively transcribed genes^[66]^. A more in-depth analysis of biological histone lysine methyltransferases has been published by Husmann and Gozani^[67]^. Counteracting these methylating efforts are KDMs such as KDM1, which demethylates H3K4 and H3K9, KDM2, which demethylates H3K36, and KDM4, which demethylates H3K9. In adult and pediatric GBM, the KDM4 subfamily of lysine demethylases retains a significant functional impact, with studies demonstrating *in vitro* elevation of KDM4A expression in the more TMZ-resistant T98G cell lines as compared to the U251MG^[68]^. Additionally, KDM2A plays an integral role in GBM immune resistance through a miR-302a/KDM2A/JAG1 axis, resulting in increased T-regulatory cell proliferation in mouse models^[69,70]^. Moreover, research by Liau *et al.* demonstrated that GSCs can enter a slow-cycling or quiescent state in response to receptor tyrosine kinase (RTK) inhibition. These slow-cycling cells depend on histone demethylase KDM6A/B, which leads to the redistribution of histone H3 lysine 27 trimethylation (H3K27me3) and contributes to tumor propagation and drug resistance^[71]^. To better contextualize clinical impact, KDMs will be further expanded upon in the analysis of therapeutic approaches.

Functionally, PRMTs serve to add methyl groups onto the arginine residues of certain target proteins, thereby disrupting their protein-protein interactions and corresponding downstream cellular processes^[16]^. Dichotomized, Type I PRMTs catalyze the mono- and asymmetric di-methylation of arginine, while Type II PRMTs catalyze mono- and symmetric di-methylation of arginine^[41]^. In GBM patients, PRMT5 and PRMT1 have historically been overexpressed and negatively associated with overall survival^[72-75]^. For example, GBM cells utilize PRMT5 to avoid mTOR inhibition, with *in vivo* inhibition of PRMT5 generally resulting in increased survival in animal models^[72,74,76]^. Furthermore, depletion of either PRMT5^[74,76,77]^ or PRMT1^[75]^ in intracranial orthotopic mouse xenograft models has demonstrated significant inhibition of tumor growth^[16]^. PRMT3 has been linked to metabolic pathway regulation in GBM, specifically preventing ubiquitination of HIF-1 to promote glycolysis. Additionally, PRMT3 knockdown in GSCs has been demonstrated to induce cell cycle arrest and apoptosis, with its inhibition causing decreased tumor growth in xenograft mouse flank models^[78]^.

#### Histone acetylation and deacetylation

Akin to their methylating counterparts, lysine acetyltransferases/histone acetyltransferases (KATs/HATs) catalyze the addition of acetyl groups to histone N-terminal lysine residues^[40]^. As mentioned previously, acetylation is believed to neutralize the charge of histone tails, thereby weakening histone-DNA and/or internucleosomal interactions^[79]^. The result is an increasingly destabilized nucleosome and chromatin structure, which allows nuclear factors such as RNA polymerase II to gain access to the DNA^[40,80,81]^. Comparatively, little work has been done in understanding the role of KATs/HATs in GBM; however, it has been identified that KAT6A is upregulated in the course of disease-promoting tumorigenesis through PIK3CA expression and the activation of the PI3K/AKT pathway^[40,82,83]^. Counteracting KATs/HATs are the histone deacetylases (HDACs), which are further subdivided into four classes based on yeast ortholog similarities. In GBM specifically, profiling experiments have highlighted significant increases in HDAC1, HDAC6, HDAC7, and HDAC10 expression and similar decreases in HDAC5 and HDAC11 expression as compared to normal brain tissue^[40,84]^. Research on individual HDACs has elucidated their contributory roles in GBM. Knockdown of HDAC1 in U87MG xenograft models correlated with a decrease in active extracellular signal-regulated kinase (ERK) and AKT, suggesting an interdependence between HDAC1 activity and the mitogen-activated protein kinase kinase (MEK)/ERK and PI3K/AKT pathways in GBM^[85]^. Moreover, HDACs have been associated with chemoradiation and TMZ resistance in GBM through their inhibition of DNA double-strand break repair^[46,86,87]^. Particularly, HDAC3 and HDAC1 overexpression in GBM is strongly associated with decreased overall survival in human patients, and its *in vitro* inhibition with RGFP109, a selective HDAC1 and HDAC3 co-inhibitor, led to greater TMZ potency in typically TMZ-resistant A172, U118, U251, and U87 cell lines^[88]^.

Additionally, acetylated lysines serve as binding sites for bromodomain-containing proteins (such as BRD4), which play crucial roles in regulating gene expression. For instance, BRD4 has been shown to enrich regions of the genome characterized by high acetylation levels, and is implicated in maintaining stem-like properties in glioblastoma cells^[89]^. Additionally, BRD4 regulates the self-renewal and tumorigenic potential of glioma-initiating cells by directly interacting with the promoter region of key genes like Notch1, further demonstrating its importance in glioma biology^[90]^. This interaction highlights how acetylation not only influences chromatin accessibility but also enables the recruitment of specific transcriptional regulators, contributing to tumor progression and resistance to therapy.

### Chromatin remodeling

Chromatin can be modified through various mechanisms involving histone modifiers, histone chaperones, and adenosine triphosphate (ATP)-dependent chromatin remodelers [Figure 1D]. These modifications alter chromatin conformation, which can either enhance or reduce its accessibility to transcription factors and the DNA repair and replication machinery. ATP-dependent chromatin remodelers, which include the switch/sucrose nonfermenting (SWI/SNF), Imitation Switch (ISWI), nucleosome remodeling and deacetylase (NuRD)/Mi-2/Chromodomain Helicase DNA-binding (CHD), INO80, and SWR1 complexes, are particularly influential in this process^[91-94]^. They orchestrate the repositioning of nucleosomes, the exchange of histone variants, and play a significant role in GBM drug resistance.

Research has shown that approximately 20% of cancers exhibit alterations in the SWI/SNF gene subunits. The SWI/SNF complex typically functions to slide and remove nucleosomes, thereby regulating chromatin structure to control gene transcription and facilitate DNA replication^[95]^. In GBM, GSCs are believed to drive sustained tumor growth, treatment resistance, and recurrence. A recent study by Di Giuseppe *et al.* investigated extracellular vesicles, particularly exosomes and microvesicles, secreted by GSCs. Their findings revealed that stimulation of the ionotropic receptor P2X7 in human GSCs led to significant proteomic changes in the released extracellular vesicles. Specifically, P2X7R activation was associated with increased glioma progression, cell aggressiveness, and migration, and it promoted the secretion of proteins that enhance therapeutic resistance, which include the chromatin remodeling protein RuvB-like 2^[96]^.

It is thought that chromatin remodelers, especially the SWI/SNF complex, are crucial in maintaining these GSC populations. Evidence suggests that the catalytic bromodomain of SWI/SNF^[97]^, BRG, is key to maintaining GSCs, with its inhibition sensitizing GSCs to TMZ and carmustine^[98]^. Another study by Ji *et al.* identified that the SWI/SNF^[99]^ subunit Actin-like 6A (ACTL6A) is highly expressed in stem and progenitor cells and supports the progenitor state. ACTL6A promotes the proliferation, invasiveness, and migration of glioma cells by regulating the YAP/TAZ pathway^[99]^. Furthermore, modifications to PFI-3, an inhibitor of the BRG1 and BRM catalytic subunits of SWI/SNF, have been shown to increase sensitization to TMZ and bleomycin^[100]^. These findings underscore the importance of understanding the chromatin remodeling mechanisms in relation to treatment resistance in GBM.

### Non-coding RNA

Non-coding RNAs (ncRNAs) are RNA molecules that are not directly translated into proteins. Still, ncRNAs are a major element of epigenetic mechanisms because of their ability to regulate gene expression. Abnormal ncRNA activity has long been associated with the regulation of tumor onset and progression for various cancers, including GBM, by acting as tumor-suppressing genes or oncogenes^[101]^. Importantly, ncRNAs have also been shown to play a critical role in GBM’s tendency to become resistant to therapeutic agents, such as TMZ chemotherapy. Several ncRNAs relevant to GBM have been identified through previous research, the dysregulation of which has already been shown to be significantly different both between GBM patients and healthy controls as well as between individuals before and after undergoing tumor resection^[102,103]^. These findings show that ncRNAs show promise both as biomarkers and as potential therapeutic targets.

There are strong associations between certain ncRNAs and different malignant characteristics of GBM, though GBM’s inherent heterogeneity means that there is some variability in whether particular ncRNAs are over- or underexpressed^[102,103]^. The ultimate impact of this dysregulation depends on the tumor microenvironment as a whole. In this context, ncRNAs have been shown to regulate angiogenesis, cell growth, cell cycle progression, apoptosis, tumor invasiveness, and immune evasion, as well as causing treatment resistance^[104]^.

Regulatory ncRNAs are separated by size. Major categories include micro-RNA (miRNA, around 20 nucleotides), short interfering RNA (siRNA, between 20 and 25 nucleotides), long non-coding RNA (lncRNA, over 200 nucleotides), and circular RNA (circRNA, over 200 nucleotides)^[105]^. The defining difference between miRNA and siRNA is that a particular miRNA may have multiple epigenetic targets while being highly specific to one RNA sequence, while siRNAs are only partially complementary to their targets and are preferred for drug discovery^[106,107]^. In both cases, the ncRNA causes gene silencing by binding to a complementary segment of an mRNA molecule, prohibiting translation. lncRNAs modulate gene transcription and the stability of mRNAs, having the potential to serve several roles as scaffolds, decoys, or transcription signal transmitters^[108]^. circRNA and lncRNAs can act as “sponges” for miRNA, binding to them and thus inhibiting the action of miRNA. circRNA molecules are similar to lncRNAs in length but take on a circular structure because their 3’ end is covalently bonded to the 5’ end^[109]^.

#### micro-RNA in glioblastoma treatment resistance

**Figure 2:**
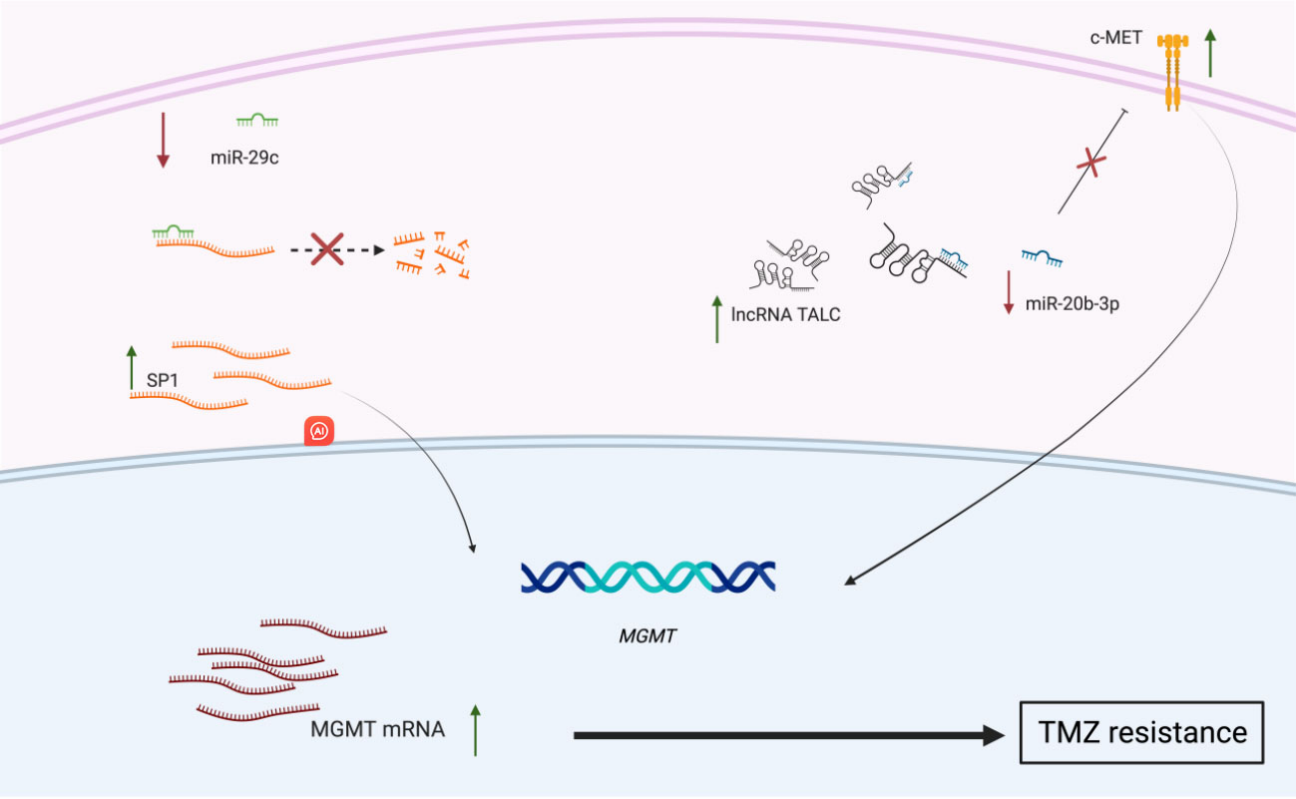
Non-coding RNAs involved in the development of temozolomide resistance in GBM. Under regular conditions, miR-29c binds to the mRNA of SP1 and induces its degradation. However, the downregulation of miR-29c in GBM leads to increased abundance in SP1 mRNA and elevated SP1 protein expression, which in turn induces the expression of the *MGMT* gene. Additionally, the overexpression of the lncRNA TALC leads to a decreased abundance of miR-20b-3p. This attenuates the inhibition that miR-20b-3p exerts over the expression of c-MET, resulting in c-MET overexpression and subsequent downstream signaling to augment *MGMT* expression. The increased *MGMT* expression induced by these mechanisms ultimately augments the GBM cells’ ability to resist treatment with TMZ. c-MET: Cellular mesenchymal-epithelial transition factor; GBM: glioblastoma; lncRNA: long non-coding RNA; mRNA: messenger RNA; *MGMT: O6-methylguanine-DNA methyltransferase*; miRNA: microRNA; SP1: specificity protein 1; TMZ, temozolomide.

Abnormal regulation of miRNAs associated with GBM leads to cascading epigenetic effects, contributing to treatment resistance (summarized in Table 1). For instance, miR-152-3p typically targets DNMT1 and the methylation of NF2, making it important for glioma apoptosis^[110]^. In GBM, miR-152-3p is downregulated, lessening glioma apoptosis. Similarly, miR-29c indirectly targets *MGMT* that supports a positive response to TMZ, and is downregulated in GBM, leading to TMZ resistance^[111]^ [Figure 2]. MiR-129-5p also targets DNMT3a and is downregulated in GBM, causing TMZ resistance and providing a good prognosis marker^[112]^. In contrast, MiR-10b-5p targets a TET2 pathway that induces tumor progression and stemness features and is upregulated in GBM^[113]^.

**Table 1:** Summary of the non-coding RNAs, along with their molecular targets, that are involved in the treatment resistance of glioblastoma

**RNA**	**Expression pattern in GBM**	**Target**	**Result**	**Reference**
**Micro RNA**				
miR-152-3p	Downregulated	DNMT1	Reduced apoptosis	[110]
miR-29c	Downregulated	*MGMT*	TMZ resistance	[111]
miR-129-5p	Downregulated	DNMT3a	TMZ resistance	[112]
miR-10b-5p	Upregulated	TET2	Tumor progression and stemness	[113]
miR-101-3p	Downregulated	EZH2	Proliferation, migration, and angiogenesis	[115]
miR-22	Downregulated	SIRT1	Proliferation, motility, and invasion	[117]
miR-9	Upregulated	PTCH1	TMZ resistance	[118]
miR-223	Upregulated	PAX6	TMZ resistance	[114,116]
miR-155-3p	Upregulated	Six1	TMZ resistance	[119]
miR-93/193	Upregulated	Cyclin D1	TMZ resistance	[120]
Long non-coding RNA				
AC016405.3	Downregulated	miR-19a-5p//TET2	Tumor progression	[121]
HOTAIR	Upregulated	PRC2 and EZH2	Cell cycle progression	[122]
LINC00461	Upregulated	miR-485-3p	Cell cycle progression	[123]
TALC	Upregulated	miR-20b-3p//*MGMT*	TMZ resistance	[124]
SNHG12	Upregulated	miR-129-5p//MAP-ERK	TMZ resistance	[125]

DNMT: DNA methyltransferases; *MGMT*: *O6-methylguanine-DNA methyltransferase*; TET: ten-eleven translocation; EZH: enhancer of zeste homolog; SIRT: silent information regulator sirtuin 1; PTCH1: patched 1; Six1: sine oculis homeobox 1; PAX6: paired box 6; MAP-ERK: mitogen-activated protein kinases- extracellular signal-regulated kinases; PRC: polycomb repressive complex.

MiR-101-3p is downregulated in GBM, but this miRNA and its targets (EZH2 and H3K27me3) have already been associated with a therapeutic strategy that targets proliferation, migration, and angiogenesis^[114-116]^. MiR-22 targets SIRT1 and its downregulation allows for tumor cell proliferation, motility, and invasion^[117]^.

Moreover, one major form of treatment resistance in GBM is resistance to TMZ. The most well-known predictor of TMZ resistance is *MGMT*. However, many miRNAs have been found to be directly involved in resistance to TMZ treatment, including miR-9 (targeting PTCH1)^[118]^, miR-223 (targeting PAX6)^[114,116]^, miR-155-3p (targeting Six1)^[119]^, and miR-93/193 (targeting Cyclin D1)^[120]^.

#### lncRNA in GBM treatment resistance

Similarly, lncRNA alterations have been widely implicated in the progression and treatment resistance of GBM (summarized in Table 1). For example, the downregulation of AC016405.3 undermines tumor suppression by disrupting DNA methylation and TET enzymes^[121]^. In this case, AC016405.3 works via miR-19a-5p to regulate TET2 and suppress tumors^[121]^. Conversely, HOTAIR is upregulated in GBM. HOTAIR targets chromatin-modifying complexes like PRC2 and regulates cell cycle progression through EZH2. Moreover, HOTAIR-targeting histone demethylase and LSD1 are upregulated cell cycle regulators that work through EZH2 to induce apoptosis^[122]^. LINC00461, another upregulated lncRNA, plays a role in GBM progression by targeting miR-485-3p which is critical to cell cycle regulation^[123]^.

There are several lncRNAs relevant to treatment resistance, such as lncRNA-TALC and SOX2OT, both of which promote TMZ resistance when upregulated. LncRNA-TALC does this by promoting *MGMT* expression and, thus, TMZ resistance^[124]^ [Figure 2]. Moreover, the upregulation of SNHG12 causes TMZ resistance through the targeting of DNA methylation of *MGMT* and even serves as a prognostic marker^[125]^.

## EPIGENETIC-BASED HETEROGENEITY AND PLASTICITY

GBM tumors are known to be among the most heterogeneous tumors classified by the 2021 World Health Organization (WHO) classification of central nervous system (CNS) tumors^[126,127]^. Heterogeneity refers to the existence of distinct subpopulations of cells within a tumor, each exhibiting diverse genotypes and phenotypes^[126,128]^. This heterogeneity seen in GBM largely contributes to its resistance to treatment. This heterogeneity is driven by the presence of a variety of epigenetic profiles that exist on the intratumoral and intertumoral levels.

When triggered by epigenetic modifications and the tumor microenvironment (TME), normal neuronal stem cells, which are normally destined to become oligodendrocytes, ependymal cells, or astrocytes, can develop into cancer stem cells (CSCs) that are referred to as GSCs^[126,129]^. One of the main features that characterizes these GSCs is their plasticity, which is defined as “morphological and functional flexibility”^[129]^. This flexibility allows GSCs to adapt to different microenvironments and persist even under harsh conditions and in the presence of therapeutic agents. In this context, plasticity also refers to a cell’s ability to interconvert from one cellular sub-state to another. This plasticity is mainly regulated by epigenetic modulations occurring in the genome, altering the expression of regulatory genes in GSCs, and driving the interchange between states in response to stimuli from the TME^[126,127]^. Herein, according to the plasticity model formulated to explain the heterogeneity within GBM and built upon the CSC model: “CSCs are the source of tumor initiation and heterogeneity…CSCs can interconvert between stem cell and differentiated states”^[130]^.

Specifically, intratumoral heterogeneity (inside of a single tumor) is maintained by epigenetic modulations, which include the previously discussed methylation of the *MGMT* gene and decreased chromatin accessibility^[130]^. Additionally, intratumoral heterogeneity is seen within GBM at the level of cellular subtypes^[131]^. Specifically, of the four subtypes identified within GSCs, the proneural (PN-GSC) and mesenchymal (Mes-GSC) subtypes are the most studied^[131]^. The PN-GSC subtype is detected in secondary GBMs and young patients, while the mesenchymal (Mes-GSC) subtype is found more often in older patients and reveals a primary and secondary GBM origin^[131]^. Mes-GSCs are known to be more aggressive phenotypes which contribute to worse prognoses in comparison to PN-GSCs. Mes-GSCs exhibit a more proliferative phenotype due to their expression of CD44, YKL40, Lyn, WT1, and BCL2A1, which are associated with angiogenesis and enhanced cell survival by counteracting apoptosis, inflammation, and cell migration/invasion^[129,130]^. In contrast, PN-GSCs express CD133, EXH2, Olig2, Sox2, and Notch1. These genes are involved in homeostasis, the cell cycle, DNA repair, and the activation of Notch and PDGF receptor signaling pathways, which impart a better prognosis^[132]^.

Compounded with genetic and epigenetic changes, the TME induces the dynamic transitions between GSCs^[127]^. In fact, heterogeneity also exists at the level of the TME and includes the hypoxic niche, the perivascular niche, and the invasive niche^[133,134]^. Herein, hypoxia is defined as a state in which oxygen is insufficient to maintain homeostasis^[135]^. Therefore, the hypoxic niche is characterized by low oxygen levels in brain tissues and the overexpression of HIFs. These factors increase the growth of HSCs and promote angiogenesis and chemoresistance^[130]^. Hypoxia offers phenotypic variability and functional characteristics to GSC subpopulations to promote adaptability to these hypoxic regions^[133]^. The invasive microenvironment, called the perivascular niche, is characterized by its collaborative network of cancer cells, endothelial cells, pericytes, astrocytes, and tumor-associated macrophages that regulate cell migration/invasion into surrounding healthy tissue^[134]^.

Hence, GBM plasticity is attained through epigenetic modifications such as histone modification, DNA methylation, and chromatin remodeling in GBM cells^[127]^. The plasticity of GBM is characterized by transitions between the proneural (less active) and mesenchymal (more active) subtypes, as well as the ability to adapt to various microenvironments. For instance, after treatment with TMZ, GSCs have been observed to adapt toward drug-resistant states, forming populations of heterogeneous drug-resistant cells. Due to the epigenetic plasticity of these cells, they are more likely to continue alternating between stem cell progenitor states and more differentiated states, depending on the prevailing conditions and microenvironment.

## THERAPEUTIC APPROACHES WITH EPIGENETIC TARGETS

This section explores the potential targeting strategies that can help attenuate the therapeutic resistance of GBM. Table 2 summarizes epigenetic modifiers that have progressed to clinical trials and describes the progress/results of these trials. ^[136,137]^

**Table 2:** Summary of the clinical trials using epigenetic modifiers, either in isolation or in combination. Based on data from clinicaltrials.gov

**Agent**	**Trials**	**Type/Design**	**Intervention**	**Status**
Valproic Acid	NCT00302159	Phase II	VPA +/- TMZ	Completed, published^[137]^
Abexinostat (PCI-24781)	NCT05698524	Phase I	Abexinostat +/- TMZ	Recruiting
Vorinostat	NCT01266031	Phase I/II	Vorinostat +/- bevacizumab	Completed, results posted on clinicaltrials.gov
	NCT02420613	Phase I	Vorinostat and temsirolimus +/- radiation therapy	Active, not recruiting
	NCT00555399	Phase I/II	Vorinostat, isotretinoin and temozolomide	Terminated, no sponsor funding for continuation of trial
	NCT01189266	Phase I/II	Vorinostat and radiation therapy	Completed, results posted on clinicaltrials.gov
	NCT00268385	Phase I	Vorinostat and temozolomide	Active, not recruiting
	NCT01738646	Phase II	Vorinostat +/- bevacizumab	Completed, results posted on clinicaltrials.gov
	NCT01378481	Phase I	Vorinostat and fractionated stereotactic body radiation therapy	Terminated
	NCT00939991	Phase I/II	Vorinostat, bevacizumab and temozolomide	Completed, results posted on clinicaltrials.gov
	NCT01236560	Phase II/III	Vorinostat, temozolomide, or bevacizumab in combination with radiation therapy followed by bevacizumab and temozolomide	Completed, results posted on clinicaltrials.gov
	NCT01110876	Phase I/II	Vorinostat, erlotinib and temozolomide	Terminated, unanticipated toxicities
	NCT00641706	Phase II	Vorinostat and bortezomib	Completed, results posted on clinicaltrials.gov
	NCT00731731	Phase I/II	Vorinostat, temozolomide, and radiation therapy	Completed, results posted on clinicaltrials.gov
	NCT00238303	Phase II	Vorinostat	Completed, results posted on clinicaltrials.gov
	NCT03426891	Phase I	Pembrolizumab and vorinostat	Completed, no results posted
	NCT00762255	Phase I	Vorinostat, bevacizumab & irinotecan	Completed, results posted^[136]^
	NCT00994500	Phase I	Vorinostat and bortezomib	Completed, no results posted
	NCT00217412	Phase I	Vorinostat +/- isotretinoin	Completed, no results posted
	NCT01076530	Phase I	Vorinostat and temozolomide	Completed, no results posted

VPA: Valproic acid; TMZ: temozolomide.

### Targeting DNA methylation

Therapeutic approaches targeting DNA methylation represent an encouraging avenue for the treatment of GBM. By reversing aberrant DNA methylation patterns associated with tumor suppressor gene silencing, these strategies aim to inhibit tumor growth, overcome therapeutic resistance, and improve outcomes for GBM patients.

DNMT inhibitors, such as azacytidine and decitabine, have been studied for their potential in treating GBM^[138,139]^. These compounds can counteract abnormal DNA hypermethylation commonly seen in GBM, resulting in the reactivation of tumor suppressor genes silenced by excessive promoter methylation^[140]^. Clinical trials are currently investigating the effectiveness of DNMT inhibitors, either alone or combined with other therapies, particularly TMZ, in managing GBM^[141]^.

Moreover, innovative genome editing techniques like clustered regularly interspaced short palindromic repeats (CRISPR)-Cas9 hold great potential for precisely modifying DNA methylation patterns in GBM. The CRISPR/Cas9 system is a type II CRISPR system that consists of three key constituents: transactivating crRNA (tracrRNA), an endonuclease (Cas9), and CRISPR RNA (crRNA). When crRNA pairs with tracrRNA, they create a molecule known as single-guide RNA (sgRNA). This sgRNA guides Cas9 to bind to the target sequence and cleave foreign DNA^[142]^. Han *et al.* used the CRISPR /Cas9 system to knock out the *MGMT* gene, thus reducing resistance to TMZ^[143]^. Similarly, to achieve the same effect of reduced TMZ resistance, Tong *et al.* used CRISPR /Cas9 to knock out the MUC1 gene^[144]^. This targeted modification could potentially reverse tumor suppressor gene silencing at specific genomic sites, paving the way for more tailored therapies for GBM patients^[142]^. Nevertheless, further investigation is necessary to refine the delivery methods and enhance the specificity of DNA methylation editing tools for their clinical implementation in GBM.

Yao *et al.* introduced a groundbreaking approach termed Methylated Oligonucleotide-Directed DNA Methylation, demonstrating its efficacy in hepatocellular carcinomas. They synthesized a methylated oligonucleotide (MON) complementary to the IGF2 promoter, inducing hypermethylation and suppressing IGF2 mRNA expression. This technique involves phosphonothioate modification to replace cytosine residues with methyl groups, resulting in 5mC formation. The MON comprises an inactivating element (IE) and a guiding element (GE). The GE directs the IE to specific loci, where a modified hemimethylated CpG hairpin structure recruits DNMT1, initiating methylation. This process mimics a replication fork, facilitating continuous DNMT1-mediated methylation. Upon dissociation, the methylated strand pairs with the unmethylated strand, creating a second hemimethylated substrate for DNMT1 to methylate, ultimately inducing site-specific gene methylation^[145]^. In a similar fashion, small non-coding RNAs can also be used to direct methylation where desired. They can guide DNA methylation by forming RNA-DNA hybrids with complementary genomic sequences through microRNA (miRNA), small interfering RNA (siRNA), or piwi-interacting RNA (piRNA) mediated pathways resulting in recruitment of DNMTs^[146]^.

Hence, identifying predictive biomarkers linked to DNA methylation patterns in GBM can help tailor treatment strategies to individual patients. Currently, the *MGMT* methylation status serves as a biomarker preceding standard chemoradiation. It is hypothesized that *MGMT* methylation status could also inform immunotherapy approaches. Evidence suggests that patients with *MGMT*-methylated tumors exhibit significantly enhanced survival rates compared to those with unmethylated *MGMT* in GBM vaccine therapy trials. Additionally, other potential biomarkers such as *PTEN* and IDH have been explored^[147-149]^. Utilizing biomarker-guided approaches enables the selection of patients who are likely to derive benefits from epigenetic therapies, thereby enhancing treatment outcomes while minimizing adverse effects. The integration of biomarker information into the clinical decision-making processes has the potential to refine the precision and efficacy of epigenetic therapies for GBM.

Another promising avenue involves targeting bromodomain and extraterminal (BET) proteins, which play a key role in regulating gene expression through their interaction with acetylated histones. Specifically, inhibition of BET proteins, such as BRD4, has been shown to sensitize glioblastoma cells to TMZ by downregulating the expression of *MGMT*, a DNA repair enzyme responsible for repairing TMZ-induced DNA damage. *MGMT* is often overexpressed in GBM and its high levels contribute to the resistance of tumor cells to TMZ therapy^[150]^.

### Targeting histone modification

#### Histone methylation and demethylation

Research into the therapeutic possibilities of histone lysine demethylase (KDM) has primarily developed over the last 15 years. In 2011, Singh *et al.*^[151]^ established the *in vitro* efficacy of KDM1 inhibition in sensitizing GBM to HDAC inhibitors and subsequent interest has led to an increase in experimental and clinical trials of KDM1 inhibitors. Inhibiting KDM1A (LSD1) with either NCL-1 or NCD-38 has been shown to decrease neurosphere formation and cell viability in GSCs, while also promoting differentiation, increasing endoplasmic reticulum stress, inducing apoptosis, and enhancing the efficacy of TMZ^[152,153]^. Additionally, another KDM1 inhibitor, tranylcypromine (TCP), combined with vorinostat, has resulted in increased apoptosis in the U87 glioma cell line *in vivo*^[151]^. Clinically, many irreversible LSD1 inhibitors have undergone cancer therapy assessment, including TCP, Iadademstat (ORY-1001), Vafidemstat (ORY-2001), GSK-2879552, Bomedemstat (IMG-7289), and INCB059872^[154]^. Due to the enduring effects of irreversible inhibitors, many reversible LSD1 inhibitors have also been researched and documented, although only two of them (Seclidemstat (SP-2577) and Pulrodemstat (CC-90011)) have progressed to clinical trials. To date, only BEA-17, a degrader of LSD1 and its cofactor, coREST, has been granted the orphan drug designation by the United States. Food and Drug Administration (FDA) for use in GBM.

Due to the previously described effects of KDM2A, it is theorized that its inhibition could augment the immunotherapeutic response against GBM, although further research is required^[46]^. This is primarily due to a lack of mechanistic understanding of KDM2A function and uncertainty regarding the *in vivo* efficacy of its inhibition. In contrast, the prevalence of the KDM4 lysine demethylase family in clinical GBM has allowed efforts to yield great strides in mechanistic understanding. In 2018, Voon *et al.* described the inhibition of KDM4 caused by pediatric GBM H3.3 G34R mutants, and the resultant epigenetic dysregulation^[155]^. Subsequent studies conducted in 2021 by Lee *et al.*^[156]^ revealed the particular significance of KDM4C in GBM tumorigenesis and p53 and c-MYC regulation. Specifically, KDM4C knockdown in U87 and U251 GBM cell lines has led to reduced colony formation, decreased c-MYC expression, and increased p53 levels. Furthermore, the inactivation of KDM5A through JIB 04, a pan-KDM inhibitor, or CP1445, a KDM5A-selective inhibitor, was found to efficiently restore TMZ sensitivity in adaptively resistant GBM cells^[46,157,158]^. When supplemented with the KDM6B inhibitor GSK-J4, JIB 04 was found to further increase potency against TMZ-resistant GBM cells^[46,158]^.

#### Histone acetylation and deacetylation

Most clinical attempts at GBM treatment through epigenetic modification have been directed toward the interplay between acetylation and deacetylation, particularly with regard to HDAC inhibitors and their ability to sensitize GBM to chemotherapeutic and radiotherapeutic treatments. Notably, almost all patients with GBM are susceptible to recurrence–a quality attributed to poor blood-brain barrier (BBB) drug permeability, intratumor heterogeneity, intrinsic GBM treatment resistance, and non-specific agent toxicities^[159,160]^. However, there are several HDAC inhibitors capable of penetrating the BBB and serving in an anti-GBM role through the upregulation of p21Waf1/Cip1, a cell-cycle inhibitor^[159]^. For example, *in vitro* treatment with the pan-HDAC inhibitor, phenylbutyrate, suppressed the proliferation of the LN-229 GBM cell line^[161]^. Accordingly, the application of romidepsin produced synergistic results in the U251MG cell line by reducing the respective anti-apoptotic protein Bcl-2^[159,162]^. Additionally, HDAC inhibitors have proven capable of regulating GBM angiogenesis through inhibition of growth factors such as VEGF and EGFR or impeding upon vascular mimicry^[159]^.

BET bromodomain inhibition (using HMBA) combined with MEK inhibition as a potential therapeutic strategy for GBM is also being explored. By targeting the BET proteins, which regulate key genes involved in tumor growth, and inhibiting the MEK-ERK signaling pathway, HMBA (BET bromodomain inhibitor) demonstrated enhanced antitumor effects compared to either approach alone. The synergistic combination led to reduced tumor cell proliferation, increased cell death, and suppression of survival pathways in preclinical models, suggesting that this dual inhibition could overcome some of the limitations of current therapies^[163]^.

Preclinically, vorinostat, suberoylanilide hydroxamic acid (SAHA), Trichostatin A (TSA), and valproic acid (VPA) have significantly precipitated GSC autophagy, reduced proliferation, and stimulated differentiation^[159,164,165]^. *In vitro* studies conducted by Urdiciain *et al.* solidified the abilities of HDAC6-selective inhibitors such as ACY-1215, tubastatin A, and CAY10603 to overturn TMZ resistance in patient-derived T98G and LN405 GBM cell lines^[166]^. In clinical trials, there have been several studies analyzing the synergistic effects of HDAC inhibitors such as vorinostat, panobinostat, and VPA with TMZ, bevacizumab, and radiation; however, none have progressed past trial phase II^[159]^. Currently ongoing are studies attempting to analyze the efficacy of various HDAC inhibitors, such as Abexinostat (PCI-24781), in the treatment of recurrent GBM. While promising, the extensive use of HDAC inhibitors within the clinical setting is limited due to a generally poor understanding of the relationship between the toxicity and pharmacokinetic properties of these inhibitors. Furthermore, identification of patients likely to respond to HDAC inhibitor treatment is difficult due to GBM heterogeneity and epigenetic profiling, compounded by the challenges of converting the promising preclinical experiments into potential therapeutic regimens for clinical trials^[159]^.

### Targeting chromatin remodeling

The dysregulation of chromatin remodeling complexes contributes to the aberrant gene expression patterns and cellular behaviors that are observed in GBM. In a recent study by Sun *et al.*, a GBM-specific epigenetic mechanism was discovered where the chromatin regulator bromodomain-contain protein 8 (BRD8) helped to maintain histone variant H2AZ at p53 targets, thereby enhancing chromatin accessibility and generating repressive chromatin state, preventing the tumor suppressor activity of p53. They found that targeting the BRD8 displaces H2AZ, allowing for the activation of p53 and leading to subsequent tumor suppression and cell cycle arrest in the TP53WT GBM cell line^[167]^. These findings suggest that targeting BRD8-mediated chromatin remolding in GBM presents a promising therapeutic strategy for overcoming the epigenetic barriers of p53 activation and potentially improving patient outcomes.

Further studies have also implicated the role of poly (ADP-ribose) polymerase (PARP) inhibitors as a potential therapeutic target in GBM. Previously, PARP1 has been implicated in the stabilization of DNA replication forks, single-stranded and double-stranded DNA breaks, as well as the modulation of chromatin structure^[168,169]^. Since PARP1 is a target of DNA damage machinery, it has been an attractive target to help generate synthetic lethality and potentiate the effects of chemotherapy and radiotherapy^[170,171]^. PARP inhibitors (PARPi), in particular Olaparib, Niraparib, and Rucaparib, have already gained approval from the FDA and have been shown to improve survival rates in patients with ovarian cancer, breast cancer, and prostate cancer, especially those with BRCA deficiencies^[172-174]^. PARPi have also been shown to play an important role in the modulation of chromatin structures that facilitate DNA repair as they will act to PARylate histone tails, resulting in a relaxed chromatin state and the removal of nucleosomes from the DNA^[168,175]^. As such, the usage of PARPi has recently garnered interest in the treatment of GBM. In the OPARTIC trial, a phase I dose escalation study of Olaparib in combination with TMZ in patients with relapsed GBM, the pharmacokinetics of Olaparib were investigated. The results showed that of the patients who received the drug, penetration was detected in 71/71 of the tumor core specimens, demonstrating its consistent ability to cross the BBB^[176]^. Therefore, the use of PARPi presents an exciting therapeutic approach with numerous other clinical trials currently evaluating their effects in the treatment of GBM.

### Targeting non-coding RNAs

#### ncRNAs as biomarkers

Some ncRNAs could be used as biomarkers to predict the progression or presence of the disease; these include miRNa-21, miRNA-26a, miR-128, and miR-342, among others. These prognosis markers could be extremely helpful in the detection and diagnosis of GBM. Early detection could reduce patient mortality and prolong survival. Additionally, other ncRNAs predict the likelihood of GBM resistance to certain treatments. In the future, this could be helpful in the clinical setting when devising treatment plans. Studies by ParvizHamidi *et al.* on miRNA-21 and miRNA-26a and by Wang *et al.* on miR-128 and miR-342 identified those miRNAs as important biomarkers^[102,103]^. Specifically, expression was quantified in people without GBM and in GBM patients before and after surgery. Before surgery, there was a large difference in the expression of these RNAs between patients with GBM and healthy subjects; however, this difference between groups was ameliorated after surgical resection. This implies that the tumor was driving the overexpression of these miRNAs, and their presence returned to normal once the tumor burden was reduced. For patients with GBM, high expression of miR-1258, miR-935, and miR-128-3p was associated with better overall survival, and the same association was found for low expression of miR-542-3p and miR-221/222^[102,103]^. These patterns could have great clinical benefits-surveying ncRNA expression in a patient could provide insights into their prognosis and possible risk of treatment resistance. This could help inform treatment plans to maximize treatment efficacy based on the individual patient’s expression patterns.

ncRNA therapies and delivery systems

The sheer number of ncRNAs means that there are many possible epigenetic targets to be explored in the therapeutic context^[177]^. Herein, miR-124-2, miR-135a-2, and let-7i were found to be the most useful miRNAs with clinical relevance in a study screening around 600 different miRNAs. Alternatively, miR-17-3p, miR-340, and miR-222 are critical miRNAs that modulate GBM cell viability *in vitro* and *in vivo*^[177]^. These two lists of relevant miRNAs do not cover the full scope of possibilities for targets of therapeutic treatment. Some other targets include several miRNAs that have been found to promote GBM sensitivity to TMZ, such as miR-198 (*MGMT*), miR-101 (GSK-3beta), miR-1268a (ABCC1), miR-381(ABCG2, ABCC3, ABCC5), miR-137 (LRP6), miR-126-3p (SOX2), and miR-128-3p (c-Met, EMT)^[178]^.

Other therapies utilize small-interfering RNA (siRNA) to target genes (such as VEGF, EGFR, STAT3, and ETDL1) that promote GBM progression and downregulate them, suppressing their oncogenic effect^[104,106]^. Other siRNAs include Livin-siRNA, which enhances TMZ sensitivity through the inhibition of MRP1, and Hsp27-siRNA, which induces apoptosis. To improve delivery efficacy, nanoparticles (NPs) are being utilized for delivery formulations. Such nanoparticles include iron oxide nanoparticles, liposomes, and other compounds specifically developed to deliver a particular siRNA^[179]^.

ncRNAs work cooperatively, and this property can be leveraged through specific combinations to enhance their overall effect. For example, the miR-Combo formulated by Bassot *et al.* used a combination of miR-17-3p, miR-222, and miR-340, which significantly decreased cell viability compared to non-targeting scrambled miRNA^[180]^.

Delivery systems

The overarching challenge in designing these therapies lies in delivering these treatments to the targets in the brain. Some obstacles to delivery include the breakdown of therapeutic agents in the blood, poor molecular stability, clearance by the kidneys, and the inability to penetrate relevant membranes. The most striking challenge in the context of GBM is the existence of the BBB^[181]^. Even if a drug can get through the BBB, it must also pass through the cell membrane to elicit any effect.

Several drug-delivery systems are being studied to improve the efficacy of GBM treatment. Some of these methods include the use of nanoparticles (NPs), which include inorganic NPs, polymeric NPs (PNPs), and lipid-based NPs (LNPs)^[104]^. NP delivery systems can provide both controlled drug release and tissue specificity while protecting miRNAs from systemic degradation, ensuring the payload remains intact. The use of synthetic RNA can increase bioavailability^[106]^, and using siRNA rather than miRNA can counteract cytotoxicity due to target specificity. Other possible delivery systems include stem cell-derived exosomes, bacterial toxins, and viral vectors to improve cell membrane penetration and successful delivery^[104]^.

## CONCLUSIONS AND FUTURE DIRECTIONS

The alterations in DNA methylation status, histone methylation and acetylation status, chromatin architecture, and expression of non-coding RNAs all play critical roles in modifying gene expression in GBM and promoting a resistant phenotype. It is imperative to study the intricate interactions between the epigenetic profiles of GBM cells and their ability to adapt to different therapeutics, along with the significant intratumoral and intertumoral heterogeneity that governs these interactions. Achieving a better understanding of these mechanisms and processes can inspire novel avenues to overcome treatment resistance and augment the efficacy of existing and emerging therapeutics. Despite a long history of investigation into epigenetic control and rewiring, further research is still required to safely and effectively navigate the transformation of these mechanisms into translatable therapies and tractable targets that can be integrated into the clinical management of GBM and offer superior outcomes to patients diagnosed with this disease. Nevertheless, it is important to note that tumorigenesis is the result of the combination of many epigenetic events. Additionally, the vast heterogeneity that is present at the intratumoral and intertumoral levels further complicates the issue. Small molecule targeting of one subset of epigenetic regulation may not lead to complete tumor eradication. The development of effective therapeutic modalities will require consideration of this complex interplay between various epigenetic events and signaling pathways that drive GBM progression.

## DECLARATIONS

### Copyright

© The Author(s) 2025.
